# Risk factors for strangulating lipoma obstruction and lipomata in horses

**DOI:** 10.1111/evj.70107

**Published:** 2025-10-04

**Authors:** Alex Gillen, Diana Hassel, Sam W. Gonzalez, Victoria Savage, Margaret Mudge, Andrew Wood, Hattie Barnes, Anje Bauck, David Freeman, Katarzyna Dembek, Liara M. Gonzalez, Debra C. Archer

**Affiliations:** ^1^ Philip Leverhulme Equine Hospital University of Liverpool Neston Cheshire UK; ^2^ College of Veterinary Medicine & Biomedical Sciences Colorado State University Fort Collins Colorado USA; ^3^ New Bolton Centre University of Pennsylvannia Kennett Square Pennsylvannia USA; ^4^ Three Counties Equine Hospital Tewkesbury Gloucestershire UK; ^5^ College of Veterinary Medicine The Ohio State University Columbus Ohio USA; ^6^ Liphook Equine Hospital Liphook Hampshire UK; ^7^ Bourton Vale Equine Clinic Cheltenham Gloucestershire UK; ^8^ College of Veterinary Medicine University of Florida Gainesville Florida USA; ^9^ College of Veterinary Medicine North Carolina State University Raleigh North Carolina USA

**Keywords:** abdominal lipoma, adiposity, colic, horse, risk factors, strangulating lipoma obstruction

## Abstract

**Background:**

Strangulating lipoma obstruction (SLO) is the most common cause of equine small intestinal strangulation and is fatal without surgery. Currently, epidemiological information is primarily limited to signalment‐related risk factors and requires further investigation.

**Objectives:**

To identify horse‐level risk factors for SLO and/or abdominal lipoma(s) (LP) formation in horses with acute colic that underwent surgery or post‐mortem examination at participating equine clinics.

**Study Design:**

Prospective, international, multicentre, epidemiological study.

**Methods:**

An epidemiological study was conducted over 27 months (January 2022–April 2024) in 8 clinics (UK *n* = 4, USA n = 4) to identify variables associated with altered likelihood of SLO and/or LP. Horses presenting with acute colic signs that underwent surgery or post‐mortem examination were eligible. Those (i) that had SLO as the primary cause of colic, or (ii) those that had mesenteric and/or omental lipoma(ta) (LP) were compared to horses without lipomata. Signalment, adiposity, endocrine status, and lipomata deposition data were analysed using univariable and multivariable logistic regression models.

**Results:**

Data from 392 horses was obtained (108 SLO; 190 LP). Increasing age (odds ratio [OR] 1.23) for every year increase in age 95% CI (95% CI: 1.17–1.30, *p* < 0.001), male sex (OR 1.78, 95% CI: 1.08–2.95, *p* = 0.02) and clinical indicators of Equine Metabolic Syndrome (EMS) (OR 4.77, 95% CI: 2.93–7.77, *p* < 0.001) were significantly associated with increased likelihood of SLO. Increasing age, clinical indicators of EMS, indicators of previous/current laminitis (hoof growth ring score), jejunal mesenteric fat score and omental fat scores were significantly associated with increased likelihood of LP.

**Main Limitations:**

Population restricted to horses with acute colic signs admitted to collaborating clinics.

**Conclusions:**

Measures to prevent adiposity and EMS development appear important to reduce the likelihood of LP and SLO. Further investigation of differential adipose tissue deposition between male and female horses is warranted.

## 1 INTRODUCTION

Strangulating lipoma obstruction (SLO) is the most common form of equine small intestinal strangulating disease, representing 7%–13.5% of horses undergoing emergency laparotomy.^1^, ^2^, ^3^, ^4^, ^5^ A challenge facing veterinary surgeons in primary care and at referral level is the early identification of horses with SLO, prior to the development of severe intestinal pathology and physiological deterioration. Knowledge of risk factors for intestinal lipomata formation and SLO can therefore help to identify at‐risk horses. Where risk factors are modifiable, strategies can also be developed to reduce the likelihood of this important form of colic.

Currently, investigation of risk factors for SLO is limited largely to signalment‐related features, and few epidemiological studies have been conducted. SLO is more likely to occur in older horses and has not been reported in horses <8 years of age.^1^, ^2^, ^6^, ^7^ Geldings also appear to be at greater risk.^1^, ^2^, ^6^ Variable and sometimes conflicting findings regarding breed‐related risk factors have been reported.^1^, ^2^, ^6^, ^8^ Epidemiological information regarding abdominal lipoma formation (LP) (defined as visible, localised fat deposition within the gastrointestinal mesentery and/or omentum) is limited to one cadaver study in which increased age was identified as a risk factor.^9^

Factors related to obesity, including greater body condition score (BCS) and weight, have been proposed as risk factors for SLO, but no evidence has been provided to support this hypothesis.^1^, ^6^ The EQUIFAT scoring system has been developed and validated as a semi‐quantitative measure of regional adiposity.^10^ Although some measures used in the EQUIFAT scoring system (epicardial fat depth and rump fat depth) can only be obtained post‐mortem, others, such as mesenteric and omental fat scores, can be measured during routine surgical exploration of the abdomen.

Endocrine disorders that are associated with obesity/insulin dysregulation (Equine Metabolic Syndrome; EMS) or increasing age (pars pituitary intermedia disorder; PPID) are plausible potential risk factors for SLO that require investigation. A cadaver study has identified increased incidence of mesenteric lipomata in horses with clinical signs of insulin dysregulation or histological evidence of pituitary hyperplasia.^9^ However, to date, these have not been evaluated as potential risk factors for SLO.

The objectives of this study were to identify horse‐level risk factors for (i) SLO and (ii) LP in a population of horses presenting with acute signs of colic that underwent emergency laparotomy or post‐mortem examination where surgical intervention was declined. A further objective was to evaluate pedunculated LP in a subgroup analysis. We hypothesised that increased age, male sex, higher adiposity scores, EMS, and PPID would be predictive of SLO and LP.

## 2 MATERIALS AND METHODS

### 2.1 Study design

A prospective, multicentre, unmatched epidemiological study was undertaken to investigate horse‐level associations with SLO and LP. Eight equine hospitals participated in the study: four in the United Kingdom (UK, Centres 1–4) and four in the United States of America (USA, Centres 5–8). Clinics were selected based on colic surgery caseload, surgical team expertise, broad geographic location, and willingness to participate in the study. Investigators underwent online training on the scoring systems used for the study (Supporting Information item 1).

Sample size calculations were performed using Stata (Intercooled Stata 18.0; StataCorp LLC). Based on a 25% exposure of EMS in controls,^11^ a study with 98 SLO horses and 294 non‐SLO horses would have 80% power to detect odds ratios of 2.0 or greater with 95% confidence. Data were collected prospectively until the required number of horses had been recruited. All horses undergoing exploratory laparotomy for the management of acute abdominal pain (colic) at participating clinics and where owner informed consent for study inclusion had been obtained were eligible. Horses in which surgery was declined and subsequently underwent post‐mortem examination following euthanasia were also eligible.

### 2.2 Study definitions

Lipoma(ta) were defined as a visible, localized and delineated/demarcated fat deposition with or without a pedicle identified within the exteriorizable gastrointestinal mesentery and/or omentum at laparotomy, or equivalent portion of mesentery when assessed at post‐mortem examination (LP). The first objective of the study was to evaluate horse‐level associations with SLO, defined as horses (horses or ponies) with acute colic undergoing laparotomy or post‐mortem examination where strangulation of bowel by a lipoma was the confirmed primary cause of acute colic. Non‐SLO were all other horses in the study where the primary cause of colic was confirmed not to be lipoma‐related. The second objective of the study was to evaluate horse‐level risk factors for formation of abdominal lipomata (LP), where lipoma(ta) were a cause of colic (SLO) or were incidental lipoma within the exteriorizable mesentery and/or omentum. Data for LP horses were compared to horses where no lipoma(ta) were evident (non‐LP) in comparable portions of the mesentery and/or omentum.

### 2.3 Data collection

Data were recorded on a specifically designed form (Supporting Information item 2). Variables evaluated included signalment (age, breed, sex), height (cm), and weight (kg). The validated EQUIFAT scoring system was used as a basis to assess and record adiposity status.^10^ Rump and epicardial adiposity could not be evaluated without post‐mortem examination and so were not included. A total modified EQUIFAT score was therefore generated. This comprised of: jejunal mesenteric fat score (grades 1–5),^10^ omental fat score (grade 1–5),^10^ retroperitoneal fat depth at the incision site (cm) measured at the midpoint of the laparotomy incision (or equivalent for post‐mortem examination), and cresty neck score (CNS) (grade 0–5)^12^ (Supporting Information item 1). Additional measures of adiposity that were also recorded included body condition score (BCS) (British Horse Society (BHS) grades 0–5)^13^ (Supporting Information item 1) and subcutaneous fat depth (cm) measured at the midpoint of the laparotomy incision (or equivalent for post‐mortem examination).

The likelihood of a horse having EMS or PPID was graded as: no clinical suspicion, mild clinical suspicion, strong clinical suspicion, and confirmed disease based on appropriate veterinary diagnostic testing, for example, baseline adrenocorticotrophic hormone.^14^ Other potential markers of endocrine status measured were supraorbital fat pad score,^12^ hoof ring divergence, and hoof ring growth scores^11^ (Supporting Information item 1). Lipomata presence within the exteriorisable mesentery of the small intestine, large and small colons, and omentum were recorded (yes/no/not evaluated) together with the number and specific morphological features of each lipoma (strangulating [SLO], incidental and pedunculated, incidental and non‐pedunculated) recorded for each horse at laparotomy/postmortem examination.

### 2.4 Data analysis

Statistical analysis was performed in Stata (Intercooled Stata 18.0; StataCorp). Variables were evaluated using a univariable logistic regression model first with SLO as the dependent variable, then with ‘any lipomata’ (LP) (SLO and incidental lipomata) as the dependent variable. Finally, horses with ‘pedunculated lipomata’ (pedunculated SLO or incidental pedunculated lipomata) were compared to horses with ‘non‐pedunculated lipomata’ (non‐pedunculated SLO or incidental non‐pedunculated lipomata) in a pre‐specified subgroup analysis. Continuous variables were assessed for normality using a Shapiro–Wilk test, and marginal analyses were utilised to assess their functional relationship with SLO and the presence of lipomata. Continuous variables were also evaluated in quintiles, quartiles, and other biologically plausible categories, with the decision for model inclusion based on model fit. The reference category for breed (Thoroughbred/Thoroughbred cross) was selected to facilitate comparison with other related studies.^2^, ^15^ Variables were screened for correlation using a Spearman or Pearson correlation coefficient, as appropriate, or, where necessary, Kendall's tau or rank biserial correlation coefficient. Where variables were considered to be measuring the same exposure or were shown to be strongly correlated (*r* > 0.7), these were selected based on biological plausibility, statistical significance, or were combined to create a composite score if appropriate. This avoided concerns regarding collinearity. Variables with a univariable *p* value <0.2 were initially selected for inclusion in a multivariable model. Missing data were recoded (imputation with a constant) to allow analysis of all horses.

Each multivariable model was built using a backwards stepwise approach. Variables were retained in a model if their manual exclusion resulted in a likelihood ratio test statistic (LRTS) of *p* < 0.05. The effect of both hospital and country was assessed in the multivariable model as predictors, confounders, and effect modifiers; they were modelled as fixed effects. Model fit was assessed using the Hosmer–Lemeshow test statistic^16^ by computing the sensitivity and specificity at various cut‐off points and by generating a receiver operating characteristic (ROC) curve. The study was reported in accordance with STROBE (The Strengthening the Reporting of Observational Studies in Epidemiology statement) reporting guidelines.^17^

## 3 RESULTS

### 3.1 Strangulating lipoma (SLO)

#### 3.1.1 Descriptive statistics

**FIGURE 1:**
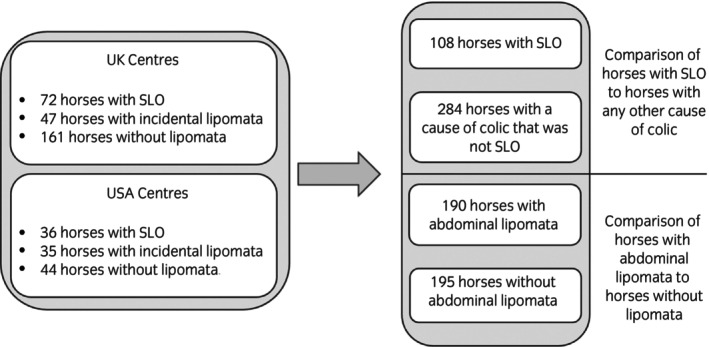
Flow chart detailing recruitment of horses onto the study from participating clinics in the United Kingdom and United States. Horses with strangulating lipoma obstruction (SLO), horses with abdominal lipomata, and horses without lipomata are shown for the United Kingdom and United States. All horses contributed to the two separate analyses performed as shown; however, 7 non‐SLO horses had incomplete information regarding the presence of incidental lipomata and were therefore excluded from this portion of the analysis.

Over the 28‐month study period, 108 cases of SLO were recruited onto the study. Two hundred and eighty‐four non‐SLO were also enrolled (Figure 1). Mean age was 18.5 years (SD 0.48) for SLO, and 11.8 years (SD 0.37) for non‐SLO. The youngest horse with SLO was aged 10. There were no cases of SLO in stallions. Mean modified EQUIFAT score for cases of SLO was 11.54 (SD 0.29), and for non‐SLO was 10.43 (SD 0.19). Non‐SLO included a variety of other primary causes of acute abdominal pain, the type and frequency of which were representative of a typical equine surgical colic population (Supporting Information item 3).

#### 3.1.2 Univariable analysis

**FIGURE 2:**
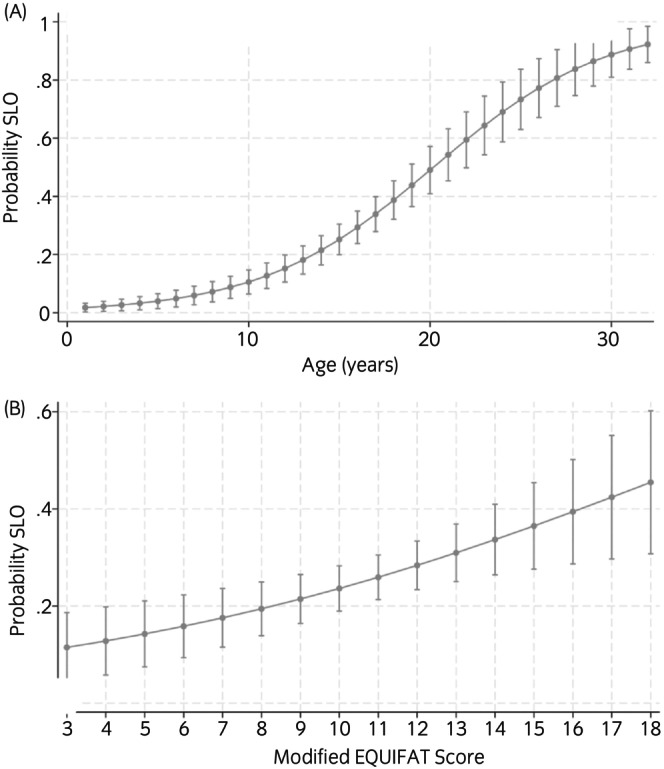
(A) Profile plot evaluating the association between age and the probability of a strangulating obstruction due to a lipoma (SLO); 95% confidence intervals are shown. (B) Profile plot evaluating the association between modified EQUIFAT score and the probability of a strangulating obstruction due to a lipoma (SLO); 95% confidence intervals are shown.

The results of the univariable logistic regression analysis, with SLO as the outcome variable, where *p* < 0.2 are shown in Table S1; variables with results of *p* < 0.05 are shown in Table 1. Evaluation of profile plots following marginal analysis for age and modified EQUIFAT score indicated that a linear fit was appropriate (Figure 2A,B). Evaluation of age and modified EQUIFAT score as continuous variables resulted in the highest significance. Age was significantly greater in SLO compared to non‐SLO horses (*p* < 0.001). Twenty‐seven female and 74 male (all geldings) had SLO; 7 horses did not have a recorded sex. Male horses (*p* = 0.02) were associated with SLO. In an attempt to evaluate the effect of geldings compared to stallions, neutered (geldings) were compared to non‐neutered (mares and stallions), with non‐neutered as the reference category. Geldings were more likely to have SLO compared to mares and stallions (OR: 2.14, 95% CI: 1.29–3.53, *p* = 0.003). Ponies (*p* < 0.001), Cobs and Welsh Section D (*p* = 0.004), and American Quarter Horses, Paint Horses and Arabians (*p* < 0.001), were all associated with SLO where Thoroughbreds and Thoroughbred crosses were used as the reference breed.

**TABLE 1:** Univariable logistic regression of 108 cases (SLO) and 284 non‐SLO horses, evaluating signalment, adiposity scores, and endocrine risk associated with the presence of strangulating lipoma (SLO).

Categorical variables					
Variable	SLO% (*n*)	Non‐SLO% (*n*)	Odds ratio	95% CI	*p* value
Breed					
TB/TBx	4.6 (5)	20.1 (57)	Reference		
WBL/ID/WBLx/IDx	13.0 (14)	21.1 (60)	2.66	0.90–7.86	0.08
Pony	25.9 (28)	15.1 (43)	7.42	2.65–20.81	<0.001
Welsh Cob/Cob	15.7 (17)	14.1 (40)	4.85	1.65–14.21	0.004
AQH/Paint/Arabian	19.4 (21)	11.3 (32)	7.48	2.57–21.75	<0.001
Other breeds	7.4 (8)	7.8 (22)	4.15	1.22–13.05	0.03
Not recorded	13.9 (15)	10.6 (30)	5.7	1.89–17.20	0.002
Sex					
Female	25.0 (27)	38.0 (108)	Reference		
Male	68.5 (74)	58.5 (166)	1.78	1.08–2.95	0.02
Not recorded	6.5 (7)	3.5 (10)	2.8	0.98–13.80	0.06
Body condition score (BCS)					
1–3	56.5 (61)	75.0 (213)	Reference		
4–5	43.5 (47)	25.0 (71)	2.31	1.45–3.68	<0.001
Cresty neck score (CNS)					
1–3	45.4 (49)	69.0 (196)	Reference		
4–6	51.9 (56)	30.6 (87)	2.57	1.62–4.07	<0.001
Not recorded	2.8 (3)	0.4 (1)	12.00	1.22–117.88	0.033
Supraorbital fat pad score					
1	52.8 (57)	75.0 (213)	Reference		
2–3	45.4 (49)	24.3 (69)	2.65	1.66–4.24	<0.001
Not recorded	1.9 (2)	0.7 (2)	3.74	0.52–27.11	0.19
PPID risk					
1	48.2 (52)	81.7 (232)	Reference		
2–4	50.9 (55)	17.3 (49)	4.92	3.01–8.02	<0.001
Not recorded	0.9 (1)	1.1 (3)	1.49	0.15–14.58	0.73
EMS risk					
1	28.7 (31)	66.2 (188)	Reference		
2–4	69.4 (75)	33.1 (94)	4.77	2.93–7.77	<0.001
Not recorded	1.9 (2)	0.7 (2)	6.06	0.23–44.65	0.08
Combined jejunal and omental fat score					
2–4	32.4 (35)	38.4 (109)	Reference		
5–10	58.3 (63)	56.7 (161)	1.25	0.77–2.03	0.36
Not recorded	9.3 (10)	4.9 (14)	2.22	0.91–15.45	0.08
Combined hoof ring score					
2	48.2 (52)	70.1 (199)	Reference		
3–6	50.0 (54)	28.5 (81)	2.55	1.61–4.04	<0.001
Not recorded	1.9 (2)	1.4 (4)	1.91	0.34–10.74	0.46

Continuous variables						
Variable	SLO not recorded % (*n*)	Non‐SLO not recorded % (*n*)	Mean	Odds ratio	95% CI	*p* value
Age (years)	12.0 (13)	6.0 (17)	13.6	1.23	1.17–1.30	<0.001
Retroperitoneal fat depth (cm)	0.9 (1)	0	2.4	1.12	0.97–1.30	0.11
Modified EQUIFAT score	13.0 (14)	5.3 (15)	10.7	1.13	1.04–1.22	0.003

Abbreviations: AQH, American Quarterhorse; EMS, Equine Metabolic Syndrome; PPID, Pars pituitary intermedia; TB/TBx, Thoroughbred/Thoroughbred Cross; WBL/ID/WBLx/IDx, Warmblood/Irish Draught/Warmblood Cross/Irish Draught Cross.

Horses with a BCS of more than 3 (with 3 being considered normal) or CNS of more than 2 (with 2 being considered normal), and a higher modified EQUIFAT score were more likely to be diagnosed with SLO (*p* < 0.001). Horses confirmed or considered likely to have PPID or EMS were more likely to have SLO, as were horses with a supraorbital fat pad score of more than 1 (*p* < 0.001). Hoof ring divergence and hoof ring prominence scores were moderately correlated (*r* > 0.7) and scores were therefore combined to create a composite score. Horses with a combined hoof ring score (addition of hoof ring divergence and hoof ring prominence scores) of more than 2 were more likely to have SLO (*p* < 0.001). No factors appeared negatively associated with SLO.

#### 3.1.3 Multivariable analysis

A final multivariable logistic regression model is shown in Table 2. In horses requiring exploratory laparotomy for colic, age had the strongest association with SLO; for each increase in age per year, the proportional likelihood of SLO increased by 23% year on year (OR: 1.23 for every year increase in age, *p* < 0.001). Male horses were more than twice as likely (OR: 2.24) to have SLO compared to female horses. A clinical suspicion of EMS was positively associated with SLO, with horses confirmed/likely to have EMS being almost 4 times more likely (OR: 3.93) to have SLO than horses where there was no clinical suspicion of SLO. The model did not show fundamental change when neutered versus non‐neutered horses were evaluated in place of male versus female horses.

**TABLE 2:** Multivariable logistic regression of signalment, adiposity scores, and endocrine risk associated with the presence of strangulating lipoma obstruction (SLO).

Variable	Odds ratio	95% CI	*p* value
Age	1.23	1.12–1.45	<0.001
Sex			
Female	Reference		
Male	2.24	1.20–4.15	0.01
EMS risk			
1	Reference		
2–4	3.93	2.19–7.06	<0.001
Not recorded	8.16	0.97–68.57	0.053

Abbreviation: EMS, Equine Metabolic Syndrome.

No significant multiplicative pairwise interactions were found between variables in the multivariable model. The Hosmer‐Lemeshow test statistic was 1.51 (*p* = 1.0, 8 degrees of freedom), indicating no evidence of poor fit. The sensitivity and specificity of the final multivariable model, with a probability cutoff of 0.5, were acceptable (49.5% and 93.2% respectively). The area under the ROC curve was 0.85, indicating that the model had very good discrimination between SLO and non‐SLO horses.^16^

### 3.2 Abdominal lipoma(ta)

#### 3.2.1 Descriptive statistics

One hundred and ninety LP (horses with any lipomata [SLO or incidental]) and 195 non‐LP (horses with no detectable lipomata) were enrolled (Figure 1). Seven non‐SLO horses could not be included in this analysis due to incomplete abdominal evaluation to assess for incidental lipomata. Ninety‐two out of 105 SLO lipomata had a pedicle (87.6%). In horses with any incidental (non‐strangulating, pedunculated, or non‐pedunculated) lipomata, 84 out of 190 (44.2%) horses had at least one pedunculated lipoma located in the abdominal cavity. The distribution of strangulating and non‐strangulating lipomata is shown in Table 3. The majority of SLO and incidental lipomata were located in the small intestinal mesentery, with 76.2% (*n* = 80) of strangulating lipomata and 65.7% (*n* = 422) of incidental lipomata being located in the small intestine.

**TABLE 3:** Table showing the intestinal location of both strangulating and non‐strangulating (incidental) lipomata (LP).

Location	Strangulating lipomata % (*n*)	Non‐strangulating lipomata % (*n*)
Omentum	9.5 (10)	11.2 (72)
Duodenum	0 (0)	0.2 (1)
Proximal third jejunum	6.7 (7)	14.0 (90)
Middle third jejunum	33.3 (35)	24.6 (158)
Distal third jejunum	22.9 (24)	19.2 (123)
Ileum	13.3 (14)	7.8 (50)
Large colon and caecum	0 (0)	8.1 (52)
Small colon	14.3 (15)	15.0 (96)
Rectum	0 (0)	0 (0)

#### 3.2.2 Univariable analysis

Variables with *p* < 0.2 in a univariable model are shown in Table S2; variables with results of *p* < 0.05 are shown in Table 4. Age was significantly greater in LP compared to non‐LP (*p* < 0.001). The youngest horse with any lipomata was 4 years of age. The youngest horse with any pedunculated lipomata (SLO in this case) was 10 years of age. All breeds were more likely to exhibit lipomata, with American Quarter Horses/Paints/Arabians being at highest risk (*p* < 0.001), using Thoroughbreds and Thoroughbred crosses as the reference breed. Sex was not associated with LP. Sixty‐three female horses and 116 male horses (2 of which were stallions) had LP; 11 cases of LP did not have a recorded sex. Sex was not related to the presence of LP, and this remained true whether sex was evaluated as male versus female, neutered versus non‐neutered, or mares compared to geldings and separately compared to stallions.

**TABLE 4:** Univariable logistic regression of 190 LP (horses with lipomata) and 195 non‐LP (horses without lipomata) evaluating signalment, adiposity scores and endocrine risk associated with presence of lipomata.

Variable	LP % (*n*)	Non‐LP % (*n*)	Odds ratio	95% CI	*p* value
Breed					
TB/TBx	7.4 (14)	23.6 (46)	Reference		
WBL/ID/WBLx/IDx	16.8 (32)	21.5 (42)	2.50	1.18–5.32	0.02
Pony	21.6 (41)	15.4 (30)	4.49	2.10–9.61	<0.001
Welsh Cob/Cob	15.3 (29)	13.9 (27)	3.53	1.59–7.81	0.002
AQH/Paint/Arabian	8.4 (16)	6.2 (17)	6.76	2.95–15.56	<0.001
Other breeds	18.4 (35)	8.7 (12)	4.38	1.68–11.42	0.003
Not recorded	12.1 (23)	10.8 (21)	3.60	1.55–8.35	0.003
Body condition score (BCS)					
1–3	36.3 (69)	76.4 (149)	Reference		
4–5	63.7 (121)	23.6 (46)	1.85	1.19–2.88	0.007
Cresty neck score (CNS)					
1–3	51.1 (97)	73.9 (144)	Reference		
4–6	47.4 (90)	25.6 (50)	2.67	1.73–4.11	<0.001
Not recorded	1.6 (3)	0.5 (1)	4.45	0.46–43.44	0.20
Supraorbital fat pad score					
1	55.8 (106)	82.1 (160)	Reference		
2–3	43.2 (82)	16.9 (33)	3.75	2.34–6.02	<0.001
Not recorded	1.1 (2)	1.0 (2)	1.51	0.21–10.88	0.68
PPID risk					
1	54.2 (103)	89.7 (175)	Reference		
2–4	44.7 (85)	9.2 (18)	8.02	4.57–14.10	<0.001
Not recorded	1.1 (2)	1.0 (2)	1.70	0.24–12.24	0.60
EMS risk					
1	34.7 (66)	75.9 (148)	Reference		
2–4	63.7 (121)	23.6 (46)	3.49	2.25–5.42	<0.001
Not recorded	1.6 (3)	0.5 (1)	6.73	0.69–65.88	0.10
Combined jejunal and omental fat score					
2–4	27.4 (52)	46.7 (91)	Reference		
5–10	66.3 (126)	48.7 (95)	2.37	1.53–3.65	<0.001
Not recorded	6.3 (12)	4.6 (9)	2.33	0.92–5.91	0.07
Combined hoof ring score					
2	47.4 (90)	79.5 (155)	Reference		
3–6	50.5 (96)	19.5 (38)	4.35	2.76–6.87	<0.001
Not recorded	2.1 (4)	1.0 (2)	3.44	0.62–19.18	0.16

Continuous variables						
Variable	Number of LP not recorded % (*n*)	Number of non‐LP not recorded % (*n*)	Mean	Odds ratio	95% CI	*p* value
Age (years)	10.0 (19)	5.6 (11)	13.6	1.25	1.19–1.32	<0.001
Retroperitoneal fat	0.5 (1)	0 (0)	2.4	1.17	1.03–1.35	0.02
Modified EQUIFAT score	8.4 (16)	5.1 (10)	10.7	1.22	1.13–1.31	<0.001

Abbreviations: AQH, American Quarter Horse; EMS, Equine Metabolic Syndrome; PPID, Pars pituitary intermedia; TB/TBx, Thoroughbred/Thoroughbred Cross; WBL/ID/WBLx/IDx, Warmblood/Irish Draught/Warmblood Cross/Irish Draught Cross.

**FIGURE 3:**
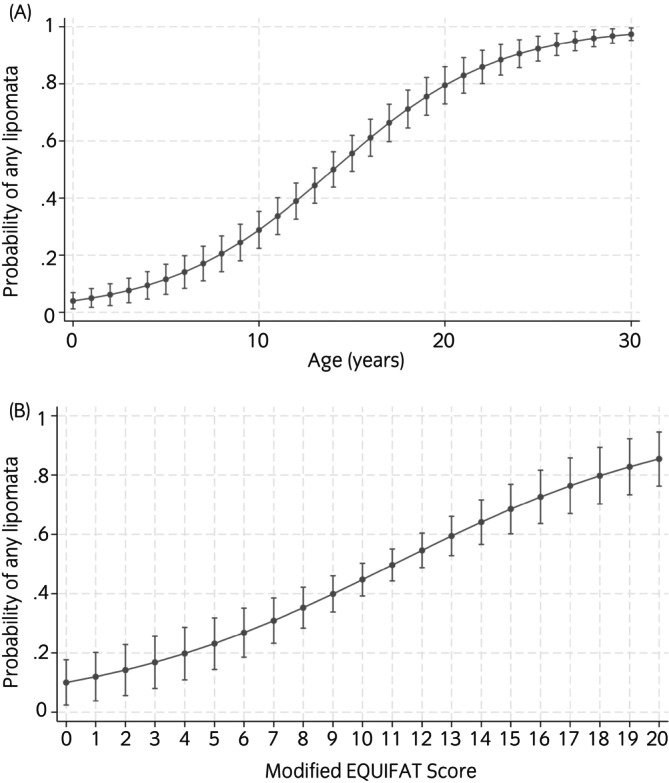
(A) Profile plot evaluating the association between age and the probability of any abdominal lipomata (LP). LP includes strangulating lipoma obstruction and incidental lipomata; 95% confidence intervals are shown. (B) Profile plot evaluating the association between modified EQUIFAT score and the probability of any abdominal lipomata (LP). LP includes strangulating lipoma obstruction and incidental lipomata; 95% confidence intervals are shown.

Horses with a BCS, or CNS, of more than 3 (BCS) or 2 (CNS) were more likely to have lipomata than those that had a BCS of 3 or less or a CNS of 2 or less (BCS: *p* = 0.007; CNS: *p* < 0.001). Increasing retroperitoneal fat depth resulted in an increased association with LP (*p* = 0.02). Due to moderate correlation (*p* > 0.7), mesenteric and omental fat scores were combined to create a composite score. A combined jejunal mesenteric and omental fat score of more than 4 (with 4 being normal) resulted in a horse being more likely to have LP than horses with a lower score (*p* < 0.001). Horses confirmed or considered likely to have PPID or EMS were more likely to have LP (*p* < 0.001). Interaction between BCS and EMS was observed (*p* = 0.04). Horses with a supraorbital fat pad score of more than 1 were more likely to have LP than horses with a supraorbital fat pad score of 1 (*p* < 0.001). A combined hoof ring score of more than 2 (grade 2 is considered normal) was more likely to exhibit LP development (*p* < 0.001). No factors were considered negatively associated with LP development in the univariable analysis. Evaluation of profile plots following marginal analysis for age and modified EQUIFAT score indicated that linear fit was appropriate (Figure 3A,B).

#### 3.2.3 Multivariable analysis

A final multivariable logistic regression model is shown in Table 5. Increasing age was strongly associated with LP, with the odds of lipomata development increasing by 1.23 for every year increase in age within this population (*p* < 0.001). A combined jejunal mesenteric and omental adipose score of more than 4 increased the likelihood of LP by 2.5 times. A clinical suspicion of EMS (OR: 3.28; *p* < 0.001) and a combined hoof ring growth score of more than 2 (OR: 2.21; *p* = 0.007) resulted in three‐ and two‐fold increases in the likelihood of LP, respectively. The model did not show any change when neutered versus non‐neutered horses were evaluated in place of male versus female horses. There was also no change when stallions and geldings were evaluated separately. Sex did not remain in the model regardless of the horse sex categories.

**TABLE 5:** Multivariable logistic regression of signalment, adiposity scores, and endocrine risk associated with the presence of lipomata (LP).

Variable	Odds ratio	95% CI	*p* value
Age	1.23	1.16–1.30	<0.001
EMS risk			
1	Reference		
2–4	3.28	1.88–5.71	<0.001
Not recorded	3.94	0.31–50.28	0.29
Combined jejunal and omental fat score			
2–4	Reference		
5–10	2.45	1.36–54.43	0.003
Not recorded	1.68	0.46–6.17	0.43
Combined hoof ring score			
2	Reference		
3–6	2.21	1.88–5.72	0.007
Not recorded	2.97	0.28–31.24	0.37

Abbreviation: EMS, Equine Metabolic Syndrome.

No significant pairwise multiplicative interactions were found between variables in the multivariable model. The Hosmer‐Lemeshow test statistic was 9.04 (*p* = 0.1715, 8 degrees of freedom), indicating no evidence of poor fit. The sensitivity and specificity of the final multivariable model, with a probability cutoff of 0.5, were good, with sensitivity and specificity being 80.7% and 77.7%, respectively. The area under the ROC curve was 0.87, indicating that the model had very good to excellent discrimination between LP and non‐LP.^16^

Sub‐group analysis of pedunculated lipomata compared to non‐pedunculated lipomata revealed that only increasing age (OR: 1.15 per year increase in age, 95% CI: 1.18–1.31; *p* > 0.001) and a clinical suspicion or confirmation of PPID (OR: 2.06, 95% CI: 1.09–3.91; *p* = 0.026) were associated with pedicle development in a univariable analysis. When evaluating horses with lipomata, the mean age of those with pedunculated lipomata was 17.9 years (SD 0.43). The mean age of horses that exhibited non‐pedunculated lipomata was 11.2 years (SD 0.39). In a multivariable analysis, only increasing age was retained (OR: 1.15 per year increase in age; 95% CI: 1.07–1.23; *p* > 0.001).

## 4 DISCUSSION

This is the first study to investigate adiposity and endocrine disorders as potential risk factors for abdominal lipomata development (LP) and lipoma‐associated colic (SLO) in horses. This international, multi‐centre study provides further evidence that age and male sex are strongly associated with SLO. The study has also identified potentially modifiable risk factors for LP and SLO. This study has shown that markers of adiposity are associated with LP within this population of horses. Prevention and effective management of EMS may help to reduce the likelihood of SLO, particularly in older, male horses.

Increased age has been identified as a risk factor for a previous history of colic in multiple studies.^18^ In previous studies, increased age was a risk factor for SLO development; control groups were horses undergoing exploratory laparotomy for colic^2^ and horses presented to a hospital for colic.^1^ In this population of horses requiring exploratory laparotomy, the likelihood of SLO and LP consistently increased with increasing age. This study confirms increasing age as being associated with both LP and the occurrence of SLO, thereby suggesting that pedunculated lipomata (representing 87.6% of SLO) may take time to develop.^15^ While previous investigations^1^, ^2^ show that increasing age is associated with the development of SLO, to the authors' knowledge, the association between increasing age and the development of LP, although suggested,^15^ has not been investigated in a prospective study in horses undergoing exploratory laparotomy. The youngest age a horse has been reported to develop SLO is 8 years of age.^2^ The youngest age of both incidental pedunculated lipomata and SLO in this study was 10 years; however, incidental non‐pedunculated abdominal lipomata were recorded in this study at 4 years of age. It is therefore plausible that lipomata deposition starts relatively early in life, but it may take several years for a lipoma to become of suitable size and weight^2^ to potentially form a pedicle, making it more likely to form SLO.

Consistent with previous studies,^1^, ^2^, ^6^ male horses in the present study were around twice as likely to develop SLO compared to female horses. It was not possible to evaluate geldings and stallions separately due to a lack of stallions with SLO; however, neutered horses were more likely to exhibit SLO than non‐neutered horses, suggesting neutering may play a role in SLO development. Conversely, the sex of the horse was not associated with LP; this remained true when neutered horses were compared to non‐neutered horses. The reasons for increased SLO in male horses, specifically geldings, have not been evaluated in detail and are an area for further investigation. However, a possible explanation may relate to hormonal changes that occur when horses are castrated. In dogs, the incidence of subcutaneous lipomata increases in neutered canines (regardless of sex) compared to non‐neutered females; this is suspected to be due to a protective effect of both male and female sex hormones.^19^ Whilst mesenteric lipomata are rare in people, both mesenteric lipomata and certain types of lipomata associated with the gastrointestinal tract occur more commonly in male compared to female patients.^20^, ^21^ In addition, reduced testosterone results in triglyceride deposition in human males.^22^ The effects of reduced testosterone should be further evaluated in geldings.

This study has shown that having any clinical evidence of EMS was associated with the development of both SLO and LP. Horses with a clinical suspicion of EMS were almost 4 times as likely to have SLO compared to horses with no clinical suspicion of EMS. Metabolism alterations and adipose dysfunction observed in horses with EMS are well‐documented.^23^, ^24^ A previous cadaver study has shown that horses with confirmed insulin dysregulation (but not increased BCS) pre‐mortem were more likely to have mesenteric lipomata at post‐mortem evaluation than horses that did not exhibit insulin dysregulation.^9^ Metabolic derangements affecting lipolysis are also exhibited in people with obesity, and this is an avenue for further research.^25^ The present study, together with post‐mortem evidence^9^ suggests that management and prevention of EMS, including weight management, appropriate exercise, testing of at‐risk horses, and appropriate medication and diet control, should be employed.^23^, ^26^ This may reduce the risk of lipomata development and therefore the risk of such lipomata becoming SLO. An intervention study would be required to determine if modification of these factors reduces the risk of SLO.

When assessing the presence of any lipomata, the combined jejunal mesenteric and omental fat score remained in the multivariable model. This suggests that markers of adiposity may play a role in the development of lipomata; however, the process by which they are able to form a strangulating lesion, such as intestinal motility^27^ or pedicle formation^15^ may involve other processes unrelated to adipose deposition. A previous study suggested, but was unable to confirm, a role of both weight and BCS in the development of SLO,^6^ however, this study suggests that these factors may be more closely linked to the development of lipomata themselves, rather than being factors that influence the ability of lipomata to form SLO. However, as SLO will only occur following the development of lipomata, weight management should be considered important.^28^ As LP were noted in horses as young as 4 years of age, weight management should commence at a young age, particularly in at‐risk breeds. However, there are several barriers to introducing weight management, particularly in breeds that traditionally have a higher BCS; therefore, owner education is essential.^28^ Factors such as the genetic and lipidomic make‐up of lipomata and adipose tissue should be further evaluated to better understand their pathogenesis in horses.^25^, ^29^ The modified EQUIFAT score, although relevant in the univariable analysis, is likely to be of limited clinical use as omental and jejunal mesenteric adipose scores can only be assessed intraoperatively.

Pars pituitary intermedia and potential indicators of this, such as an increased supraorbital fat pad and elevated hoof growth ring scores, were all predictive of the development of both LP and SLO in the univariable analysis but did not remain in the multivariable model for SLO. An increased total hoof growth ring score remained in the multivariable model for LP. Although PPID is typically associated with older horses,^14^ this study found only a mild correlation between age and a clinical suspicion of PPID in a population undergoing exploratory laparotomy or post mortem examination for abdominal pain. A post mortem study has shown that horses with diffuse pituitary hyperplasia, and therefore presumed pituitary dysfunction, had an increased incidence of mesenteric lipomata; this was irrespective of insulin status.^9^ However, confirmed/suspected PPID did not remain in the multivariable models. A combined hoof ring divergence and hoof ring prominence score was associated with LP in the present study. Although this study did not specifically evaluate laminitis, hoof ring scores can be indicative of current or previous laminitis (within the last 5 years).^11^ This may indicate a period of uncontrolled PPID or EMS, and this is being investigated in a further study; however, a causal pathway between PPID and EMS has not been established.

Breed did not remain in either multivariable analysis; however, the results of the univariable analyses are in agreement with other studies where ponies^2^ and Arabians^6^ were found to be at increased risk of development of SLO compared to Thoroughbreds and Thoroughbred crosses. The reason for breed not remaining in the multivariable analysis may be partially due to the fact that EMS is more common in breeds such as ponies and cobs.^11^, ^26^ In addition, body condition score can be related to breed,^30^ and ponies are considered at increased risk of being overweight compared to other breeds.^31^ Therefore, breed may be a marker for increased likelihood of EMS and increased fat deposition. This suggests that weight management and prevention of EMS are essential in at‐risk breeds.

Limitations of this study include the fact that the study was conducted on horses undergoing exploratory laparotomy for abdominal pain, thereby introducing population bias as not all horses with colic are presented to a referral hospital.^32^, ^33^ The risk of PPID or EMS was determined by clinician assessment of each horse's physical appearance. In addition, the methods of testing for confirmed PPID or EMS cases were not documented. Therefore, it is possible that not all horses with diagnosed PPID or EMS will have undergone appropriate testing. Although clinician training regarding EMS and PPID took place, bias is possible; agreement between clinicians and consistency in scoring between clinicians was not assessed. All exploratory laparotomy cases during the study period at Centre 1 were enrolled and were entered into the INCISE database (INCISE International Colic Audit: https://internationalcolicaudit.com), allowing reporting of the lesion in non‐SLO horses. This may not have been the case with other centres, where SLO and non‐SLO enrolment depended on the attending surgeon being a study collaborator, reducing the ability to compare the study population to the overall exploratory laparotomy population. However, evaluation of the population of non‐SLO horses in Centre 1 showed a range of both large and small intestinal cases.

In conclusion, the likelihood of the development of LP is related to age, adiposity, and markers of endocrine disease. SLO is more common in older male horses with a clinical suspicion of EMS, and this should be considered by the veterinary surgeon when evaluating colic cases. This study suggests that LP and the occurrence of SLO could be reduced by adopting strategies to control adiposity, protect against laminitis, and manage EMS. LP can occur in horses as young as 4 years of age, and the introduction of management strategies to reduce risk early in life is recommended. The associated factors differ between the development of LP and SLO; therefore, further investigation into lipidomics and adipose metabolism is recommended to further establish risk factors for the development of SLO.

## FUNDING INFORMATION

Arden and Claudia Sims Lipoma Foundation.

## CONFLICT OF INTEREST STATEMENT

None.

## AUTHOR CONTRIBUTIONS

**Diana Hassel:** Investigation; visualization; project administration; supervision; writing – review and editing. **Sam W. Gonzalez:** Investigation; visualization; writing – review and editing. **Victoria Savage:** Investigation; visualization; project administration; writing – review and editing. **Margaret Mudge:** Investigation; visualization; project administration; supervision; writing – review and editing. **Andrew Wood:** Investigation; visualization; project administration; writing – review and editing. **Hattie Barnes:** Investigation; visualization; project administration; writing – review and editing. **Anje Bauck:** Investigation; visualization; project administration; writing – review and editing. **David Freeman:** Investigation; visualization; project administration; supervision; writing – review and editing. **Katarzyna Dembek:** Investigation; visualization; project administration; writing – review and editing. **Liara M. Gonzalez:** Investigation; visualization; project administration; supervision; writing – review and editing. **Alex Gillen:** Conceptualization; investigation; funding acquisition; writing – original draft; methodology; validation; visualization; software; formal analysis; project administration; data curation; resources; writing – review and editing. **Debra C. Archer:** Conceptualization; investigation; funding acquisition; writing – original draft; visualization; writing – review and editing; formal analysis; supervision; resources.

## DATA INTEGRITY STATEMENT

Alexandra Gillen and Debra Archer had full access to all the data in the study and take responsibility for the integrity of the data and the accuracy of the data analysis.

## ETHICAL ANIMAL RESEARCH

Ethical approval for the study at UK centres was granted by the University of Liverpool (VREC 1154) and/or the RCVS. US centres underwent ethical approval through their universities. IACUC approval was not required for US centres.

## INFORMED CONSENT

Explicit owner consent for inclusion of animals in this study was not obtained. Owners or their agents were made aware that case information may be used for research in general.

## Supporting information


**Table S1:** Univariable logistic regression of 108 SLO (strangulating lipoma obstruction) and 284 non‐SLO horses, evaluating signalment, adiposity scores and endocrine risk associated with presence of strangulating lipoma (SLO).

**Table S2:** Univariable logistic regression of 190 LP (horses with lipomata) and 195 non‐LP (horses without lipomata) evaluating signalment, adiposity scores and endocrine risk associated with presence of lipomata.

**Data S1:** **Supporting Information Item 1:** Scoring systems.

**Data S2:** **Supporting Information Item 2:** Lipoma study scoresheet.

**Data S3:** **Supporting Information Item 3:** Pie chart and table showing the distribution of lesion type amongst controls at Centre 1.

## Data Availability

The data that support the findings of this study are available upon reasonable request from the corresponding author. Open data sharing exemption granted by the editor.
