# Supplementary material for: Risk factors for strangulating lipoma obstruction and lipomata in horses
Source: Equine Vet J. 2025 Oct 4;58(4):1005–15. doi: 10.1111/evj.70107 (PMC13244193; doi:10.1111/evj.70107)
Supplement: Supplementary file 1 — Table S1: Univariable logistic regression of 108 SLO (strangulating lipoma obstruction) and 284 non‐SLO horses, evaluating signalment, adiposity scores and endocrine risk associated with presence of strangulating lipoma (SLO). [file EVJ-58-1005-s003.pdf]

Table S1: Univariable logistic regression of 108 SLO (strangulating lipoma obstruction) and 284 non-SLO horses, evaluating signalment, adiposity scores and endocrine risk associated with presence of strangulating lipoma (SLO).

Categorical Variables

Variable	SLO % (n)	Non-SLO % (n)	Odds Ratio	95% CI	P Value
**Breed**					
**TB/TBx**	4.63 (5)	20.07 (57)		Reference	
**WBL/ID/WBLx/IDx**	12.96 (14)	21.13 (60)	2.66	0.90-7.86	0.08
**Pony**	25.93 (28)	15.14 (43)	7.42	2.65-20.81	<0.001
**Welsh Cob/Cob**	15.74 (17)	14.08 (40)	4.85	1.65-14.21	0.004
**AQH/Paint/Arabian**	19.44 (21)	11.27 (32)	7.48	2.57-21.75	<0.001
**Other breeds**	7.41 (8)	7.75 (22)	4.15	1.22-13.05	0.03
**Not recorded**	13.90 (15)	10.56 (30)	5.7	1.89-17.20	0.002
**Sex**					
**Female**	25.00 (27)	38.03 (108)		Reference	
**Male**	68.52 (74)	58.45 (166)	1.78	1.08-2.95	0.02
**Not recorded**	6.48 (7)	3.52 (10)	2.8	0.98-13.80	0.06
**Country**					
**UK**	66.67 (72)	77.82 (221)		Reference	

**US**	33.33 (36)	22.18 (63)	1.75	1.07-2.86	0.03
**Centre**					
**1**	48.12 (52)	55.28 (157)		Reference	
**2-4**	18.51 (20)	22.54 (64)	0.44	0.52-1.71	0.85
**5**	12.96 (14)	9.86 (28)	1.51	0.74-3.08	0.26
**6-8**	20.37 (22)	12.32 (35)	1.90	1.02-3.52	0.04
**Body condition score (BCS)**					
**1-3**	56.48 (61)	75.00 (213_		Reference	
**4-5**	43.52 (47)	25.00 (71)	2.31	1.45-3.68	<0.001
**Cresty neck score (CNS)**					
**1-3**	45.37 (49)	69.01 (196)		Reference	
**4-6**	51.85 (56)	30.63 (87)	2.57	1.62-4.07	<0.001
**Not recorded**	2.78 (3)	0.35 (1)	12.00	1.22-117.88	0.033
**Supraorbital fat pad score**					
**1**	52.78 (57)	75.00 (213)		Reference	
**2-3**	45.37 (49)	24.30 (69)	2.65	1.66-4.24	<0.001
**Not recorded**	1.85 (2)	0.70 (2)	3.74	0.52-27.11	0.19
**PPID risk**					
**1**	48.15 (52)	81.69 (232)		Reference	
**2-4**	50.93 (55)	17.25 (49)	4.92	3.01- 8.02	<0.001
**Not recorded**	0.93 (1)	1.06 (3)	1.49	0.15-14.58	0.73

**EMS risk**					
**1**	28.70 (31)	66.20 (188)		Reference	
**2-4**	69.44 (75)	33.10 (94)	4.77	2.93-7.77	<0.001
**Not recorded**	1.85 (2)	0.70 (2)	6.06	0.23-44.65	0.08
**Combined jejunal and omental fat score**					
**2-4**	32.41 (35)	38.38 (109)		Reference	
**5-10**	58.33 (63)	56.69 (161)	1.25	0.77- 2.03	0.36
**Not recorded**	9.26 (10)	4.93 (14)	2.22	0.91-15.45	0.08
**Combined hoof ring score**					
**2**	48.15 (52)	70.07 (199)		Reference	
**3-6**	50.00 (54)	28.52 (81)	2.55	1.61-4.04	<0.001
**Not recorded**	1.85 (2)	1.41 (4)	1.91	0.34-10.74	0.46

TB/TBx = Thoroughbred/Thoroughbred Cross
WBL/ID/WBLx/IDx = Warmblood/Irish Draft/Warmblood Cross/ Irish Draft Cross
AQH = American Quarterhorse
EMS = Equine metabolic syndrome
PPID = Pars pituitary intermedia dysfunction

Continuous Variables

Variable	SLO not recorded % (n)	Non-SLO not recorded % (n)	Mean	Odds Ratio	95% CI	P Value

**Age (years)**	12.04 (13)	5.99 (17)	13.6	1.23	1.17-1.30	<0.001
**Retroperitoneal fat depth**	0.93 (1)	0	2.4	1.12	0.97-1.30	0.11
**Modified EQUIFAT score**	12.96 (14)	5.28 (15)	10.7	1.13	1.04-1.22	0.003
