# Supplementary material for: Risk factors for strangulating lipoma obstruction and lipomata in horses
Source: Equine Vet J. 2025 Oct 4;58(4):1005–15. doi: 10.1111/evj.70107 (PMC13244193; doi:10.1111/evj.70107)
Supplement: Supplementary file 2 — Table S2: Univariable logistic regression of 190 LP (horses with lipomata) and 195 non‐LP (horses without lipomata) evaluating signalment, adiposity scores and endocrine risk associated with presence of lipomata. [file EVJ-58-1005-s001.pdf]

Table S2: Univariable logistic regression of 190 LP (horses with lipomata) and 195 non-LP (horses without lipomata) evaluating signalment, adiposity scores and endocrine risk associated with presence of lipomata.

Variable	LP % (n)	Non-LP % (n)	Odds Ratio	95% CI	*P* Value
**Breed**					
**TB/TBx**	7.37 (14)	23.59 (46)		Reference	
**WBL/ID/WBLx/IDx**	16.84(32)	21.54 (42)	2.50	1.18-5.32	0.02
**Pony**	21.58 (41)	15.38 (30)	4.49	2.10-9.61	<0.001
**Welsh Cob/Cob**	15.26 (29)	13.85 (27)	3.53	1.59-7.81	0.002
**AQH/Paint/Arabian**	8.42 (16)	6.15 (17)	6.76	2.95-15.56	<0.001
**Other breeds**	18.42 (35)	8.72 (12)	4.38	1.68-11.42	0.003
**Not recorded**	12.11 (23)	10.77 (21)	3.60	1.55-8.35	0.003
**Country**					
**UK**	66.84 (127)	82.05 (160)		Reference	
**US**	33.16 (63)	17.95 (35)	2.28	1.41-3.64	0.001
**Centre**					
**Centre 1**	52.11 (99)	53.33 (104)		Reference	
**Centres 2-4**	14.81 (28)	28.72 (56)	0.44	0.52-1.71	0.85
**Centre 5**	13.23 (25)	8.72 (17)	1.51	0.74-3.08	0.26
**Centres 6-8**	20.11 (38)	9.23 (18)	1.90	1.02-3.52	0.04
**Body condition score (BCS)**					
**1-3**	63.68 (121)	76.41 (149)		Reference	
**4-5**	36.32 (69)	23.59 (46)	1.85	1.19-2.88	0.007
**Cresty neck score (CNS)**					
**1-3**	51.05 (97)	73.85 (144)		Reference	
**4-6**	47.37 (90)	25.64 (50)	2.67	1.73-4.11	<0.001
**Not recorded**	1.58 (3)	0.51 (1)	4.45	0.46-43.44	0.20
**Supraorbital fat pad score**					
**1**	55.79 (106)	82.05 (160)		Reference	
**2-3**	43.16 (82)	16.92 (33)	3.75	2.34-6.02	<0.001
**Not recorded**	1.05 (2)	1.03 (2)	1.51	0.21-10.88	0.68
**PPID risk**					
**1**	54.21 (103)	89.74 (175)		Reference	
**2-4**	44.74 (85)	9.23 (18)	8.02	4.57-14.10	<0.001
**Not recorded**	1.05 (2)	1.03 (2)	1.70	0.24-12.24	0.60
**EMS risk**					
**1**	34.74 (66)	75.90 (148)		Reference	
**2-4**	63.68 (121)	23.59 (46)	3.49	2.25-5.42	<0.001
**Not recorded**	1.58 (3)	0.51 (1)	6.73	0.69-65.88	0.10

**Combined jejunal and omental fat score**					
**2-4**	27.36 (52)	46.67 (91)		Reference	
**5-10**	66.32 (126)	48.72 (95)	2.37	1.53-3.65	<0.001
**Not recorded**	6.32 (12)	4.62 (9)	2.33	0.92-5.91	0.07
**Combined hoof ring score**					
**2**	47.37 (90)	79.49 (155)		Reference	
**3-6**	50.53 (96)	19.49 (38)	4.35	2.76-6.87	<0.001
**Not recorded**	2.11 (4)	1.03 (2)	3.44	0.62-19.18	0.16

TB/TBx = Thoroughbred/Thoroughbred Cross
WBL/ID/WBLx/IDx = Warmblood/Irish Draft/Warmblood Cross/ Irish Draft Cross
AQH = American Quarter Horse
EMS = Equine Metabolic Syndrome
PPID = Pars pituitary intermedia

Continuous Variables

Variable	Number of LP not recorded	Number of non-LP not recorded	Mean	Odds Ratio	95% CI	*P* Value
**Age (years)**	19	11	13.7	1.25	1.19-1.32	<0.001
**Height (cm)**	9	9	152.4	0.99	0.98-1.00	0.09
**Retroperitoneal fat depth (cm)**	1	0	2.4	1.17	1.03-1.35	0.02
**Modified EQUIFAT score**	16	10	10.7	1.22	1.13-1.31	<0.001
