# Supplementary material for: Risk factors for strangulating lipoma obstruction and lipomata in horses
Source: Equine Vet J. 2025 Oct 4;58(4):1005–15. doi: 10.1111/evj.70107 (PMC13244193; doi:10.1111/evj.70107)
Supplement: Supplementary file 3 — Data S1: Supporting Information Item 1: Scoring systems. [file EVJ-58-1005-s002.pdf]

Equine Veterinary Journal BEVA

Supplementary item 1: Body condition scoring system

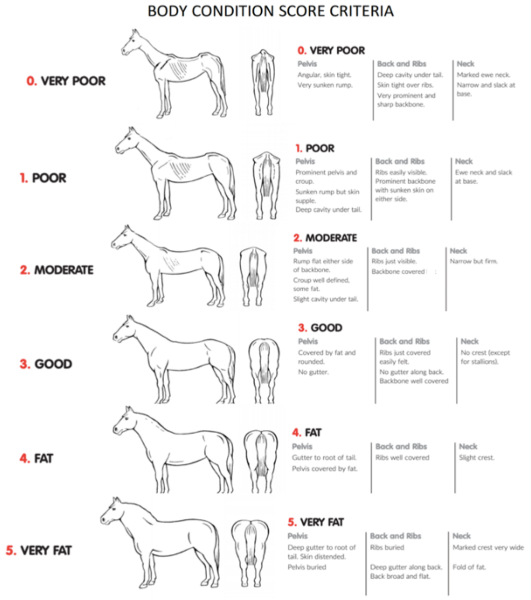

Society BH: Fat Scoring. https://wwwbhsorguk/horse-care-and-welfare/health-care-management/horse-health/fat-scoring/, 2024.

Supplementary item 1: Cresty neck score

CRESTY NECK SCORE

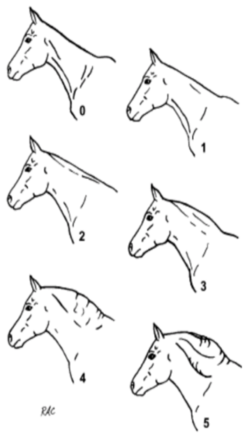

Score	Description
0	No visual appearance of crest (tissue apparent above the *ligamentum nuchae*. No palpable crest
1	No visual appearance of crest, but slight filling felt with palpation
2	Noticeable appearance of crest, but fat deposited fairly evenly from poll to withers. Crest easily cupped in one hand and bend from side to side
3	Crest enlarged and thickened, so fat is deposited more heavily in middle of the neck than toward poll and withers, giving a mounded appearance. Crest fills cupped hand and begins losing side to side flexibility
4	Crest grossly enlarged and thickened, and can no longer be cupped in one hand or easily bend from side to side. Crest may have wrinkles/ creases perpendicular to topline
5	Crest is so large it permanently droops to one side

Carter RA, Geor RJ, Burton Staniar W, et al: Apparent adiposity assessed by standardised scoring systems and morphometric measurements in horses and ponies. Vet J 179:204-210, 2009.

Supplementary item 1: Supraorbital fat pad grade

Grade of supraorbital fat pad

1: The soft tissue within the supraorbital fossa is concave relative to the surrounding bone

2: The soft tissue within the supraorbital fossa is level with the surrounding bone

3: The soft tissue within the supraorbital fossa is convex relative to the surround bone

Carter RA, Geor RJ, Burton Staniar W, et al: Apparent adiposity assessed by standardised scoring systems and morphometric measurements in horses and ponies. Vet J 179:204-210, 2009.

Supplementary item 1: Hoof ring divergence and hoof ring prominence scores

Hoof Growth Ring Divergence

Grade	Description	Example
1	All growth rings are parallel to the coronary band, or show only very mild separation towards the heel.	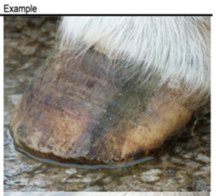
2	Toward the heel, growth rings show moderate separation but remain straight and/or show mild distal deviation	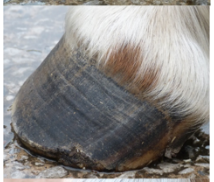
3	Towards the heel, growth rings show marked separation and/or marked distal deviation	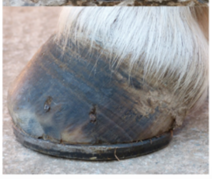

Hoof Growth Ring Prominence

Grade	Description	Example
1	Hoof capsule is smooth, or growth rings only just visible or palpable	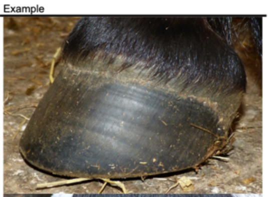
2	Moderate depth growth rings clearly visible and palpable	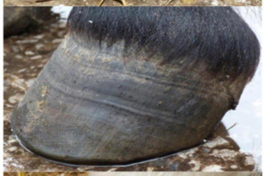
3	Prominent, deep growth rings visible	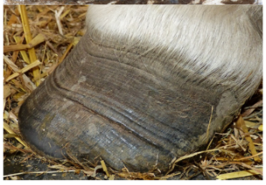

Carter RA, Geor RJ, Burton Staniar W, et al: Apparent adiposity assessed by standardised scoring systems and morphometric measurements in horses and ponies. Vet J 179:204-210, 2009.

Supplementary item 1: Lipoma obstruction type

Lipoma Obstruction Type

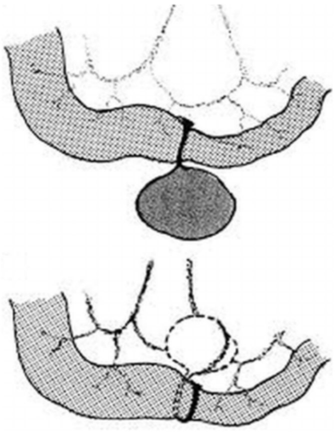

Type A: Lipoma creates a single band around the intestine

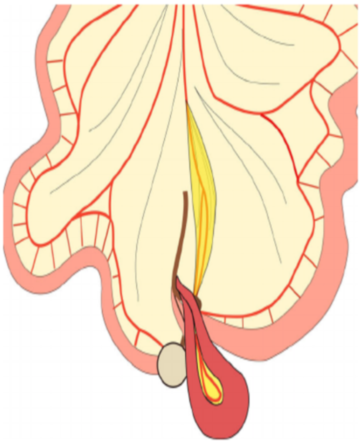

Type B: Lipoma entraps a loop of intestine

Type C: Other

Supplementary item 1: Mesenteric and omental fat grade

MESENTERIC FAT<br>Evaluate ~30cm of mesentery extending distally from the serosal margin of a ~0.5 m loop of proximal jejunum.		
**Score**	**Descriptor**	**Exemplar**
**1**	No or minimal fat visible.	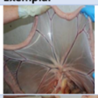
**2**	Fat in the immediate vicinity of the superior mesenteric vessels (SMVs) but arterial arcades still clearly visible.	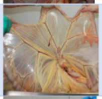
**3**	Distinct fat deposits around and beginning to fill the spaces between SMVs. SMVs partially obscured by fat.	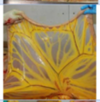
**4**	Extensive accumulations of fat largely obscuring and filling spaces between most arcades of the SMVs.	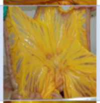
**5**	Mesenteric peritoneum, SMVs completely obscured by fat.	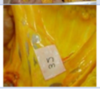

OMENTAL FAT<br>Evaluate ~30cm of omentum extending distally from the midpoint of the greater curvature of the stomach		
**Score**	**Descriptor**	**Exemplar**
**1**	No or minimal fat visible	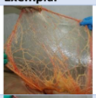
**2**	Fat in immediate vicinity of the gastroepiploic vessels (GEVs) but vessels still clearly visible.	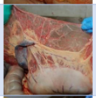
**3**	Distinct fat deposits around and beginning to fill the spaces between GEVs. GEVs partially obscured by fat.	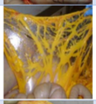
**4**	Extensive accumulations of fat largely obscuring and filling the spaces between most GEVs.	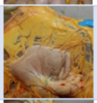
**5**	Omental peritoneum and GEVs completely obscured by fat.	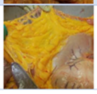

Morrison PK, Harris PA, Maltin CA, et al: EQUIFAT: A novel scoring system for the semi-quantitative evaluation of regional adipose tissues in Equidae. PLoS One 12:e0173753, 2017.
