# Supplementary material for: Risk factors for strangulating lipoma obstruction and lipomata in horses
Source: Equine Vet J. 2025 Oct 4;58(4):1005–15. doi: 10.1111/evj.70107 (PMC13244193; doi:10.1111/evj.70107)
Supplement: Supplementary file 4 — Data S2: Supporting Information Item 2: Lipoma study scoresheet. [file EVJ-58-1005-s005.pdf]

SUPPLEMENTARY ITEM 2: LIPOMA STUDY – ADJUNCT DATA TO INCISE COLIC AUDIT

HORSE NAME: ☐ **CLINIC HORSE ID:** ☐ **DATE:** ☐

L1. HEIGHT AT WITHERS: ☐ HANDS or ☐ CM

L2. BODY CONDITION SCORE (PLACE CROSS IN BOX)

0. VERY POOR	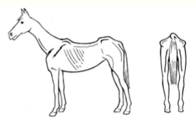	☐	2. MODERATE	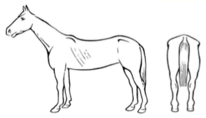	☐	4. FAT	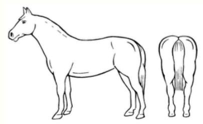	☐
1. POOR	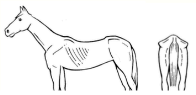	☐	3. GOOD	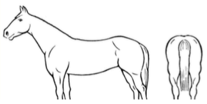	☐	5. VERY FAT	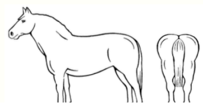	☐

L3. CRESTY NECK SCORE (place cross in box)

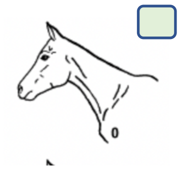 ☐	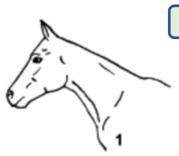 ☐	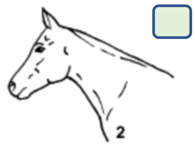 ☐	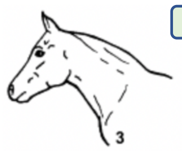 ☐	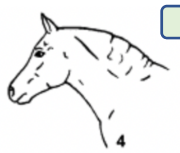 ☐	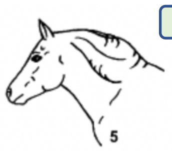 ☐
0	1	2	3	4	5

L4. SUPRAORBITAL FAT PAD SCORE (place 'X' in relevant box)

The soft tissue within the supraorbital fossa is concave relative to the surrounding bone		The soft tissue within the supraorbital fossa is level with the surrounding bone		The soft tissue within the supraorbital fossa is convex relative to the surrounding bone	

L5. HOOF RING DIVERGENCE (place 'X' in relevant box)

All growth rings parallel to coronary band or only very mild separation towards heels		Moderate separation of hoof rings towards heel but remain straight / mild distal deviation		Marked separation and / or marked distal deviation of growth rings towards heel	

L6. HOOF RING PROMINENCE SCORE (place 'X' in relevant box)

Hoof capsule is smooth or growth rings only just visible / palpable		Moderate depth growth rings clearly visible and palpable		Prominent, deep growth rings visible	

L7. EQUINE METABOLIC SYNDROME STATUS (place 'X' in relevant box)

EMS confirmed by testing		Highly likely has EMS but not confirmed		Possible clinical signs of EMS		No clinical signs of EMS, extremely unlikely	

L8. CUSHINGS / PPID STATUS (place 'X' in relevant box)

PPID Confirmed by testing		Highly likely has PPID but not confirmed		Possible clinical signs of PPID		No clinical signs of signs PPID, extremely unlikely	

L9. RETROPERITONEAL FAT DEPTH (cm) ☐ **L10. SUBCUTANEOUS FAT DEPTH (cm)** ☐

L11. DOES THE HORSE HAVE A LIPOMA CAUSING A GASTROINTSTINAL OBSTRUCTION?

YES ☐ *PLEASE COMPLETE BELOW* **NO** ☐ *(GO TO NEXT PAGE)*

LOCATION (please circle)	PEDICLE LENGTH (cm)	TYPE OF OBSTRUCTION*	Lipoma SIZE (CMxCM)	LENGTH OBSTRUCTED (CM)	OBSTRUCTED GUT VIABLE? Y / N	LENGTH RESECTED (cm)
Proximal 1/3 jejunum<br>Middle 1/3 jejunum<br>Distal 1/3 jejunum<br>Ileum<br>Small colon<br>Omentum<br>Other: (State)<br>Unsure (snapped off)						000<br><br>N/A

L12. OMENTAL FAT SCORE

Evaluate ~30cm of mesentery extending from serosal margin of a 0.5cm section of proximal jejunum	
Score	Descriptor
1 ☐	No / minimal fat visible
2 ☐	Fat around Superior mesenteric vessels (SMVs) but arterial arcades clearly visible
3 ☐	Fat deposits around and beginning to fill spaces between SMVs. SMVs partially obscured by fat
4 ☐	Extensive accumulations of fat largely obscuring and filling spaces between most arcades of SMVs
5 ☐	Mesenteric peritoneum and SMVs completely obscured by fat

L13. JEJUNAL MESENTERIC FAT SCORE

Evaluate ~30cm of omentum extending distally from the mid point of the greater curvature of the stomach	
Score	Descriptor
1 ☐	No / minimal fat visible
2 ☐	Fat around gastroepiploic vessels (GEV's) but other vessels clearly visible
3 ☐	Fat deposits around and beginning to fill spaces between GEVs. GEVs partially obscured by fat
4 ☐	Extensive accumulations of fat largely obscuring and filling spaces between most GEVs
5 ☐	Omental peritoneum and GEVs completely obscured by fat

L14. PRESENCE / ABSENCE OF OMENTAL / MESENTERIC LIPOMAS (for obstructing lipomas complete Q.11)

Location	Lipomas present (place number or X in box)			Size (cm x cm)	Pedicle length (cm)	Sessile (S) / Pedunculated(P)
	Yes (number)	No	Not assessed			
**Omentum**	00					
**Duodenum**	00					
**Proximal 1/3rd Jejunum**	00					
**Mid 1/3rd jejunum**	00					
**Distal 1/3rd jejunum**	00					
**Ileum**	00					
**Caecum**	00					
**Large colon**	00					
**Small colon**	00					
**Rectum**	00					
**Additional:** State location						
