# Supplementary material for: Risk factors for strangulating lipoma obstruction and lipomata in horses
Source: Equine Vet J. 2025 Oct 4;58(4):1005–15. doi: 10.1111/evj.70107 (PMC13244193; doi:10.1111/evj.70107)
Supplement: Supplementary file 5 — Data S3: Supporting Information Item 3: Pie chart and table showing the distribution of lesion type amongst controls at Centre 1. [file EVJ-58-1005-s004.pdf]

Equine Veterinary Journal BEVA

Supplementary item 3: Pie chart and table showing the distribution of lesion type amongst controls at Centre 1. The lesion types are well distributed and are representative of the hospital colic caseload.

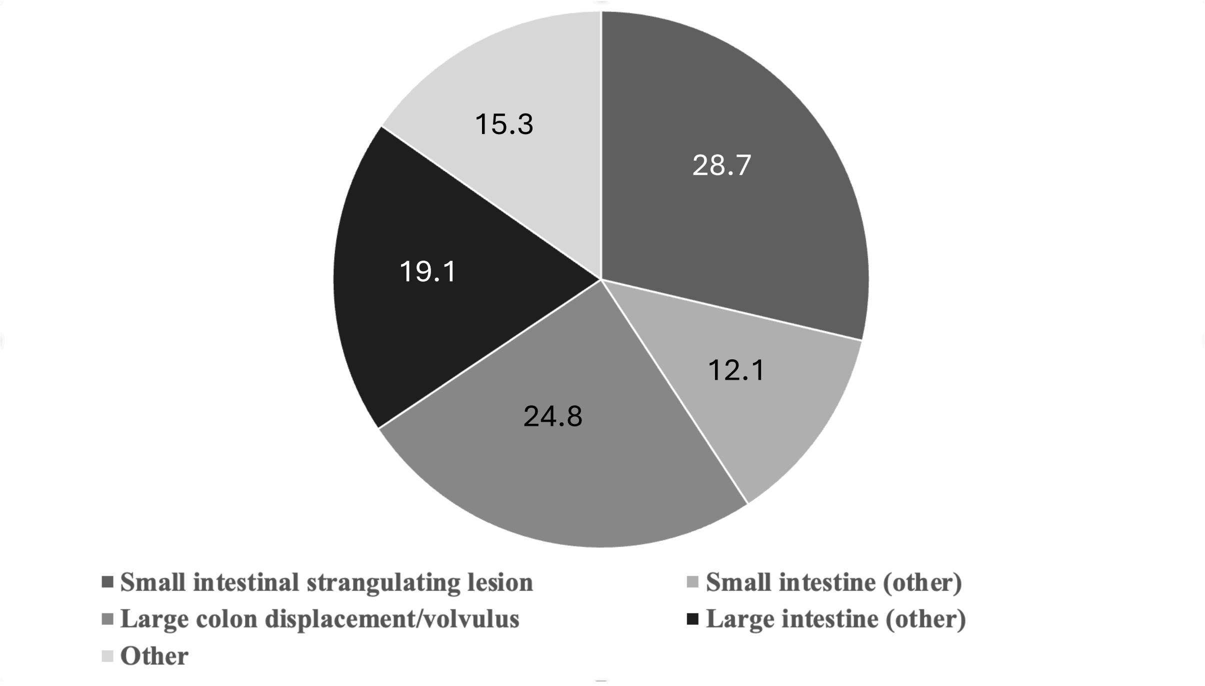

Lesion Type	Percentage (n)
Small intestinal strangulating lesion	28.7 (45)
Small intestine (other)	12.1 (19)
Large colon displacement/volvulus	24.8 (39)
Large intestine (other)	19.1 (30)
Other	15.3 (24)
